# JAK1 Pseudokinase V666G Mutant Dominantly Impairs JAK3 Phosphorylation and IL-2 Signaling

**DOI:** 10.3390/ijms24076805

**Published:** 2023-04-06

**Authors:** Alice H. Grant, Alejandro C. Rodriguez, Omar J. Rodriguez Moncivais, Shengjie Sun, Lin Li, Jonathon E. Mohl, Ming-Ying Leung, Robert A. Kirken, Georgialina Rodriguez

**Affiliations:** 1 Department of Biological Sciences, The University of Texas at El Paso, El Paso, TX 79968, USA; 2 Border Biomedical Research Center, The University of Texas at El Paso, El Paso, TX 79968, USA; 3 Department of Physics, The University of Texas at El Paso, El Paso, TX 79968, USA; 4 Computational Science Program, The University of Texas at El Paso, El Paso, TX 79968, USA; 5 Department of Mathematical Sciences, The University of Texas at El Paso, El Paso, TX 79968, USA

**Keywords:** JAK1, JAK3, allosteric inhibitor, pseudokinase, JH2, phosphorylation, IL-2

## Abstract

Overactive Janus kinases (JAKs) are known to drive leukemia, making them well-suited targets for treatment. We sought to identify new JAK-activating mutations and instead found a JAK1-inactivating pseudokinase mutation, V666G. In contrast to other pseudokinase mutations that canonically lead to an active kinase, the JAK1 V666G mutation led to under-activation seen by reduced phosphorylation. To understand the functional role of JAK1 V666G in modifying kinase activity we investigated its influence on other JAK kinases and within the Interleukin-2 pathway. JAK1 V666G not only inhibited its own activity, but its presence could inhibit other JAK kinases. These findings provide new insights into the potential of JAK1 pseudokinase to modulate its own activity, as well as of other JAK kinases. Thus, the features of the JAK1 V666 region in modifying JAK kinases can be exploited to allosterically inhibit overactive JAKs.

## 1. Introduction

Janus kinases (JAKs) are at the forefront of cytokine signaling, working immediately downstream of cytokine receptors, which lack intrinsic catalytic activity. The JAK family consists of four tyrosine kinases (JAK1, JAK2, JAK3, and tyrosine protein kinase 2 (TYK2)), and through paired combinations with seven signal transducers and activators of transcription (STATs), they orchestrate cell-specific cytokine signaling events that ultimately dictate patterns of gene expression [1]. JAK homo- and hetero-dimerization is critical in the propagation of the signal transduction cascade by tyrosine phosphorylation [2,3,4]. JAK1 and JAK3 proteins associate with their respective cognate receptors. Specifically, the common gamma chain (γc) receptor recruits JAK3 [5,6]. γc-linked receptors (γc-LR) respond to Interleukin-2 (IL-2), IL-4, IL-7, IL-9, IL-15, and IL-21 [3,5,6]. These latter cytokines regulate cellular processes including cell differentiation, growth, proliferation, and survival. Many of these functions are accomplished by the classic IL-2 cytokine. JAK1 and JAK3 associated with the IL-2 pathway have revealed separate roles for each JAK. JAK1 is recognized for initiating Tyr trans-phosphorylation with JAK3 in response to IL-2 [7], whereas JAK3 harbors the bulk phosphorylation of Tyr residues residing along IL-2Rβ chain [8], creating docking sites for SH2 containing proteins that initiate downstream pathways. In the JAK/STAT pathway, STAT5 binds IL-2Rβ and undergoes Tyr phosphorylation mediated by JAK1. In fact, JAK3 dimerized with the JH1 mutated kinase-dead JAK1 K908A inhibits STAT5 phosphorylation [9]. Thus, JAK1 is necessary for JAK3 activation and downstream IL-2 signaling. Mutations in JAK1, JAK3 or γc can result in hyper- or hypo-cellular activities. In leukemia, hyperactive JAK family members, notably JAK1, JAK2, and JAK3, are recognized as oncogenic drivers.

Aggressive forms of acute lymphoblastic leukemia (ALL) are believed to be driven by mutated JAKs [10,11] or to indirectly activate the JAK/STAT pathway [12]. Overactive JAK/STAT signaling possibly contributes to 10.7% of all high-risk ALL cases [13]. Currently, the Food and Drug Administration (FDA)-approved JAK inhibitors are being tried against pediatric ALL [14]. These drugs represent a significant effort to combat refractory or relapse ALL [15] where the long-term overall survival rate is 15–50% [16]. While JAK inhibitors have been approved for the treatment of atopic dermatitis, myelofibrosis, polycythemia vera, graft vs. host disease, rheumatoid arthritis, idiopathic juvenile arthritis, psoriasis, ulcerative colitis, and ankylosing spondylitis [17], none have been approved for the treatment of ALL. Clinical studies support their efficacy [18], yet treatment is associated with adverse events, including anemia and thrombocytopenia [19]. Adverse events are reflective of off-target effects [20], resulting from their promiscuity for binding multiple JAKs. This is because ATP mimetics [21] are the classic JAK inhibitors approved by the FDA.

The majority of pipeline JAK inhibitors target the ATP-binding site and are classified as Type I and Type II inhibitors, depending on whether the kinase is active or inactive, respectively. High conservation of the ATP-binding site among the JAK family and across human kinases allows such inhibitors to act broadly. Although these pleotropic effects by current JAK inhibitors may be beneficial in shutting down multiple signaling pathways utilized by cancer cells, this feature may also be contributing to the unwelcome adverse effects that have raised concerns for their clinical use [22]. To overcome this issue, new strategies are needed that target JAKs in a selective manner. Type III and Type IV inhibitors work allosterically, either proximal or distal to the ATP-binding site, respectively [23]. These distal regions are likely not entirely conserved, which is ideal for capturing JAK selectivity. In fact, the clinically selective allosteric TYK2 inhibitor was recently approved by the FDA for the treatment of moderate-to-severe plaque psoriasis [24,25]. Nevertheless, there are limited allosteric JAK inhibitors available which have been approved by the FDA [26]. Thus, new allosteric strategies to inhibit JAKs are needed, aside from the current FDA-approved JAK inhibitors, which have yet to reach their full potential in treating ALL.

The structure of JAKs consists of four functional domains harboring seven Janus Homology (JH) motifs, including the carboxyl-terminal JH1 Tyr kinase domain and the JH2 pseudokinase domain. The amino-terminal end houses the 4.1, Ezrin, Radixin, Moesin (FERM) domain and the Src Homology 2 (SH2) domain, jointly spanning JH3–JH7. The bulk of activating mutations in JAK family members occurs within functional “hot spots” [27] found across the JH domains, the most notable occurring in JH2. For example, in high-risk Philadelphia chromosome (Ph)-like B-ALL, the JAK1 V658F mutation occurs in JH2 [28]. Similarly, in T-ALL, JAK3 contains high frequency pseudokinase domain mutations, including the M511I variant [29] in the SH2–JH2 linker and A573V in JH2 [30]. The nature of these activating JH2 mutations stems from the idea that the JH2 pseudokinase domain provides negative regulation against the JH1 enzymatic domain. Thus, strategies to harness the intrinsic inhibition by the JH2 in contrast to exploiting the JH1 active site is a promising avenue for inhibiting JAK kinases allosterically.

Initially, we sought to identify novel activating JAK mutations driving ALL, but instead found an inactivating JAK1 JH2 mutation and explored its potential in modifying kinase activity. Specifically, in vitro kinase assays revealed that the V666G mutation resulted in a JAK1 kinase-dead-like phenotype. A dominant negative effect of inactive JH1 and JH2 JAK1 mutants occurred on wild-type (WT) JAK family members, as observed by changes in Tyr phosphorylation. The JAK1 V666 region can modify its own intrinsic catalytic activity and that of dimerized JAK2 and JAK3 kinases as well. Perhaps most interesting, JAK1 V666G decreased Tyr phosphorylation of the ALL-associated JAK3 constitutively active mutants, M511I and A573V. These findings demonstrate that JAK1 V666G exerts a dominant negative role on JAK3. Likewise, these findings support the notion of shared trans-inhibitory interactions between the JAK1 JH2 and hyperactive JAK3 JH1, suggesting that the V666 belt of the JAK1 pseudokinase may be exploited by therapies that aim to inhibit overactive JAKs in an allosteric manner.

## 2. Results

### 2.1. Identification of the JAK1 V666G Mutation

**Figure 1:**
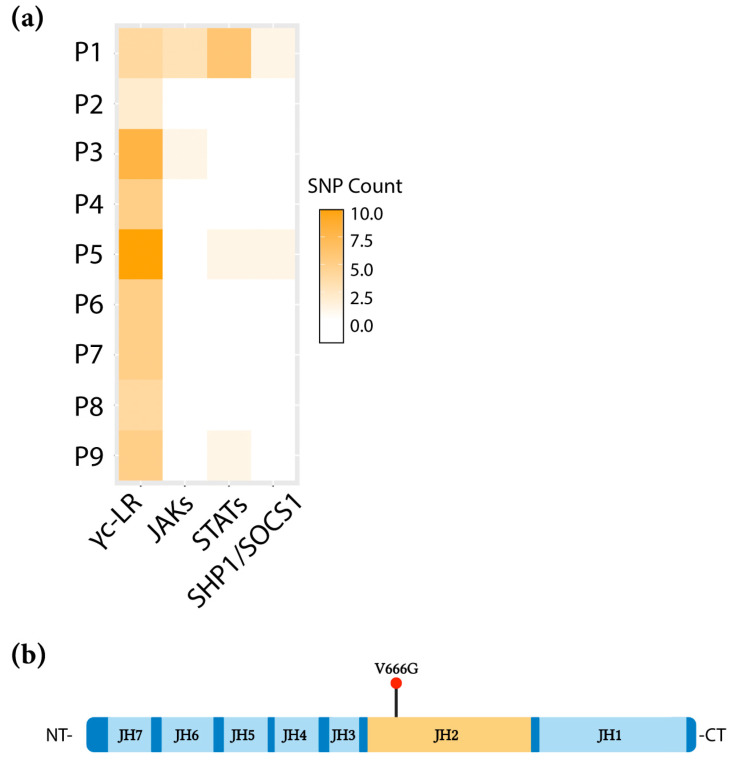
Identification of the JAK1 V666G mutation in an ALL-patient sample. (**a**) The number of non-synonymous ALL SNPs within genes involved in the JAK/STAT pathway across the patient cohort (P1–P9) are represented in a heat map. Common gamma-chain-linked receptors (γc-LR), Janus tyrosine kinases (JAKs), signal transducer and activator of transcription (STAT), SH-2 containing inositol 5′ polyphosphatase 1 (SHIP1), suppressor of cytokine signaling 1 (SOCS1). (**b**) Linear structure of JAK1, depicting V666G within the JH2 domain (orange). Figure was created with BioRender.com, accessed on 2 February 2023.

Nine ALL patient (P1-P9) specimens, analyzed by whole exome sequencing (WES) and reported previously [31], containing single-nucleotide polymorphisms (SNPs) occurring in JAK/STAT pathway genes, and not present in controls, are depicted in a heat map (Figure 1a) to represent the frequency of mutations within each individual sample. All nine patients contained a high frequency of γc-linked receptor (γc-LR) mutations (Figure 1a, represented by tones of orange). Multiple specimens, P3, P5, and P9, had mutations in various JAK/STAT-pathway-related genes. Non-synonymous JAK or STAT mutations were not detected in P2, P4, and P6–P8 samples (Figure 1a, represented by white boxes). P1 diagnosed with pre-T ALL displayed a higher number of JAK/STAT mutations, including the frequently observed activating STAT5B N642H mutation [31,32]. The aim was to identify novel activating JAK/STAT mutations that were unreported with a PROVEAN score of less than −2.5 and located in a domain of interest. P1 contained an unreported JAK1 valine (V) 666-to-glycine (G) heterozygous mutation, which is conserved in JAKs, had a PROVEAN score of −6.846, and a variant allele frequency (VAF) score at 20% (Supplementary Figure S1). A search of the Catalogue of Somatic Mutations in Cancer (COSMIC) and Therapeutically Applicable Research to Generate Effective Treatments (TARGET) databases supported its absence in the literature. The JAK1 V666G mutation was of interest particularly because of the location of the residue within the JH2 pseudokinase domain (Figure 1b), a “hot spot” for gain-of-function mutations [33].

### 2.2. Abrogation of JAK1 V666G Auto-Activation and Substrate Phosphorylation

**Figure 2:**
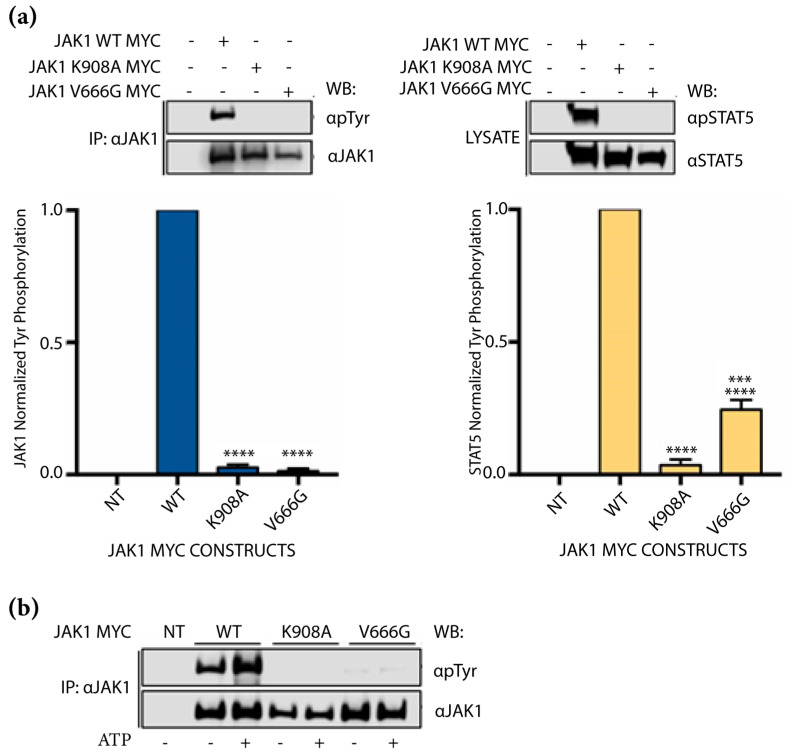
Abrogation of JAK1 V666G auto-activation and substrate phosphorylation. Western blot (WB) of protein from U4C cells non-transfected (NT, –/–/–) or transiently transfected to overexpress either JAK1 WT, or the JAK1 MYC constructs, kinase-dead JAK1 K908A or JAK1 V666G with STAT5. Representative blots are shown with quantified phosphorylation normalized to total protein, using densitometry (bar graphs). (**a**) Overexpressed JAK1 was immunoprecipitated (IP) to assess endogenous autoactivation indicated by JAK1 Tyr phosphorylation (blue bars). Corresponding input total cell lysate was used to assess JAK1 phosphorylation of STAT5 (yellow bars). Graphed data represent the means ± SEM of normalized Tyr phosphorylation from independent experiments (*n* = 3). Statistical significance, *** *p* <0.0005 and **** *p* < 0.0001, was determined using a one-way ANOVA with a post hoc Tukey’s test for multiple comparisons. (**b**) Immunoprecipitated JAK1 was challenged by an in vitro kinase assay in the presence or absence of 100 μM ATP for 20 min, and blotted for Tyr phosphorylation. Representative data of *n* = 3 independent experiments are shown.

To investigate the impact of V666G on JAK1 kinase activity, auto-activation via overexpression in the JAK1-deficient cell line, U4C, was coupled to in vitro kinase assays, and Tyr phosphorylation of JAK1 and STAT5 were measured. Auto-activation is defined as the phosphorylation that occurs between the same JAK kinases and is not facilitated by cognate receptors. Compared to JAK1 WT, auto-activation of JAK1 V666G was similar to that of the kinase-dead control, JAK1 K908A, with a significant observable decrease in Tyr phosphorylation (Figure 2a, left panel). Additionally, JAK1 V666G Tyr phosphorylation of STAT5 was decreased, similar to the kinase-dead protein (Figure 2a, right panel). Employing in vitro kinase assays, JAK1 V666G displayed reduced Tyr phosphorylation when compared to WT protein in the presence of ATP (Figure 2b). Thus, in contrast to other common JAK1 pseudokinase mutants in ALL, the JAK1 V666G mutation, identified herein, displayed hypoactivation.

### 2.3. JAK1 V666G Inhibits Cross-Activation of JAK1, JAK2, and JAK3 Dimer Partners

**Figure 3:**
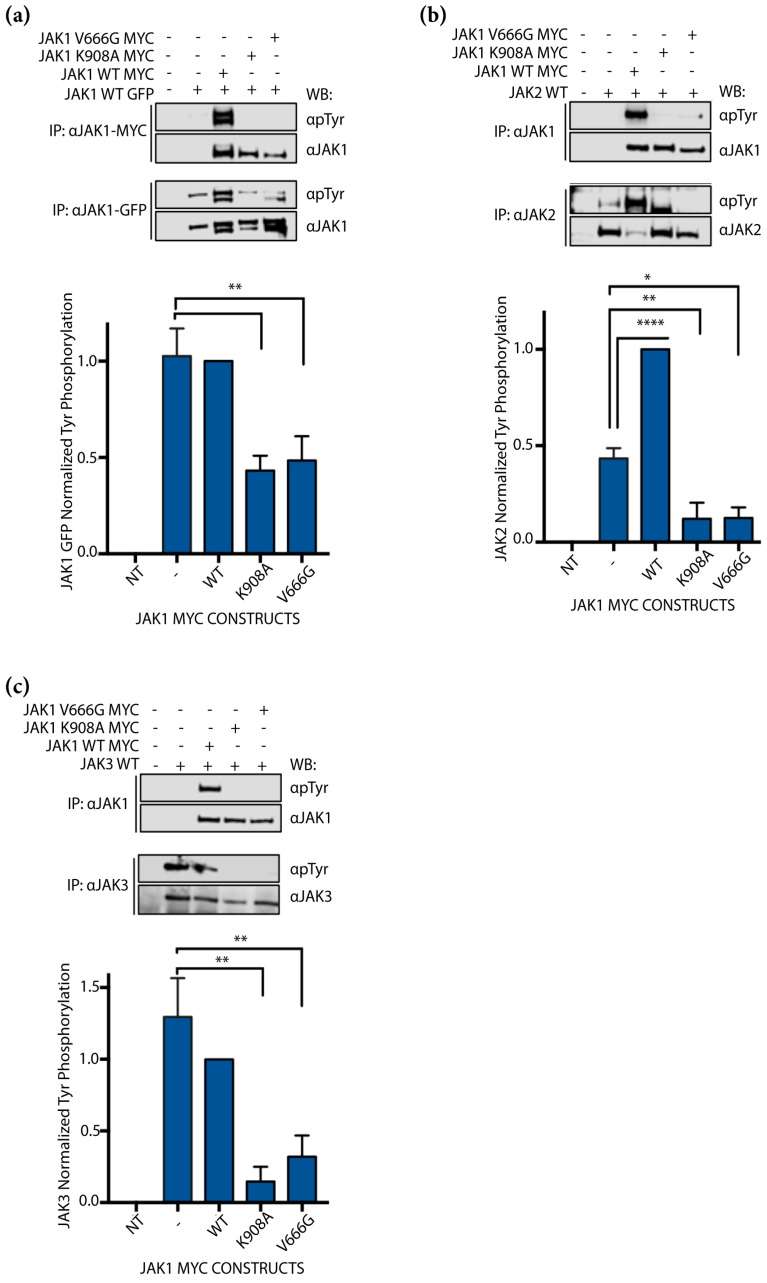
JAK1 V666G inhibits cross-activation of partner JAK1, JAK2, and JAK3. Western blot (WB) of protein lysates from U4C cells non-transfected (NT, –/–/–/–) or transiently transfected to overexpress JAK constructs. Representative blots are shown with quantified Tyr phosphorylation normalized to total protein using densitometry, and plotted with the mean and ± SEM from no fewer than three independent experiments (*n* ≥ 3) (blue bars). (**a**) WT JAK1–GFP was overexpressed with or without WT JAK1–MYC, JAK1 K908A, or JAK1 V666G, and immunoprecipitated (IP) for MYC and GFP tags. JAK1 cross-activation, indicated by Tyr phosphorylation, was assessed by WB. Graphed data represent WT JAK1 GFP-normalized Tyr phosphorylation (*n* = 4). Statistical significance, ** *p* < 0.0062, was determined using a one-way ANOVA with a post hoc Tukey’s test for multiple comparisons. (**b**) WT JAK2 was overexpressed with or without JAK1–MYC WT, JAK1 K908A, or JAK1 V666G, immunoprecipitated for JAKs, and subsequently assessed for cross-activation, indicated by Tyr phosphorylation. Graphed data represent JAK2-normalized Tyr phosphorylation, (*n* = 3). * *p* < 0.0101, ** *p* < 0.0092, **** *p* < 0.0001. (**c**) JAK3 WT was overexpressed with or without either JAK1–MYC WT, JAK1 K908A, or JAK1 V666G, immunoprecipitated for JAKs, and subsequently assessed for cross-activation, indicated by Tyr phosphorylation. Graphed data represent JAK3-normalized Tyr phosphorylation (*n* = 3). ** *p* < 0.0055.

To investigate whether JAK1 V666G could be activated by WT JAK family members, JAK1–MYC constructs were co-expressed with either WT JAK1–GFP, JAK2 or JAK3, and examined for cross-activation indicated by Tyr phosphorylation of both JAKs. Cross-activation here is defined as the phosphorylation between distinct JAK kinases not facilitated by cognate receptor proteins. Similar to kinase-dead JAK1 K908A, JAK1–MYC V666G was not phosphorylated by JAK1–GFP WT protein (Figure 3a). However, in the presence of MYC-tagged kinase-dead JAK1 K908A and JAK1 V666G, Tyr phosphorylation of WT JAK1–GFP (Figure 3a, lower panel) was significantly decreased compared to samples with JAK1 WT protein. There was no statistical significance found between Tyr phosphorylation of JAK1–GFP samples with and without JAK1–MYC WT protein (Figure 3a). Similarly, JAK1–MYC V666G was not phosphorylated by WT JAK2. In the presence of JAK1 K908A and JAK1 V666G, Tyr phosphorylation of JAK2 WT protein was also significantly decreased compared to samples with WT JAK1 (Figure 3b). Tyr phosphorylation of JAK2 was also significantly greater in the presence of WT JAK1 compared to assays without JAK1. Lastly, JAK1–MYC V666G was not phosphorylated by JAK3 WT protein (Figure 3c). Again, in the presence of JAK1 K908A and JAK1 V666G, Tyr phosphorylation of WT JAK3 was significantly decreased compared to samples with WT JAK1 (Figure 3c). JAK3 without JAK1 was Tyr phosphorylated to the same extent as JAK3 in the presence of WT JAK1. Taken together, neither JAK1 WT, JAK2 WT nor JAK3 WT proteins were capable of phosphorylating JAK1 V666G. Furthermore, WT JAK proteins expressed without a partner JAK MYC construct were capable of auto-activation (Figure 3a–c). In all instances JAK1 WT, JAK2 WT, and JAK3 WT were statistically greater in Tyr phosphorylation in the presence of JAK1 WT protein than when in the presence of inactive JAK1 K908A or V666G constructs. Hence, the presence of classic JAK1 kinase-dead JH1 mutation or JH2, V666G mutation can hinder auto- and cross-activation in vitro.

### 2.4. JAK1 V666G Inhibits Trans-Activation of JAK3 and IL-2 Signaling

**Figure 4:**
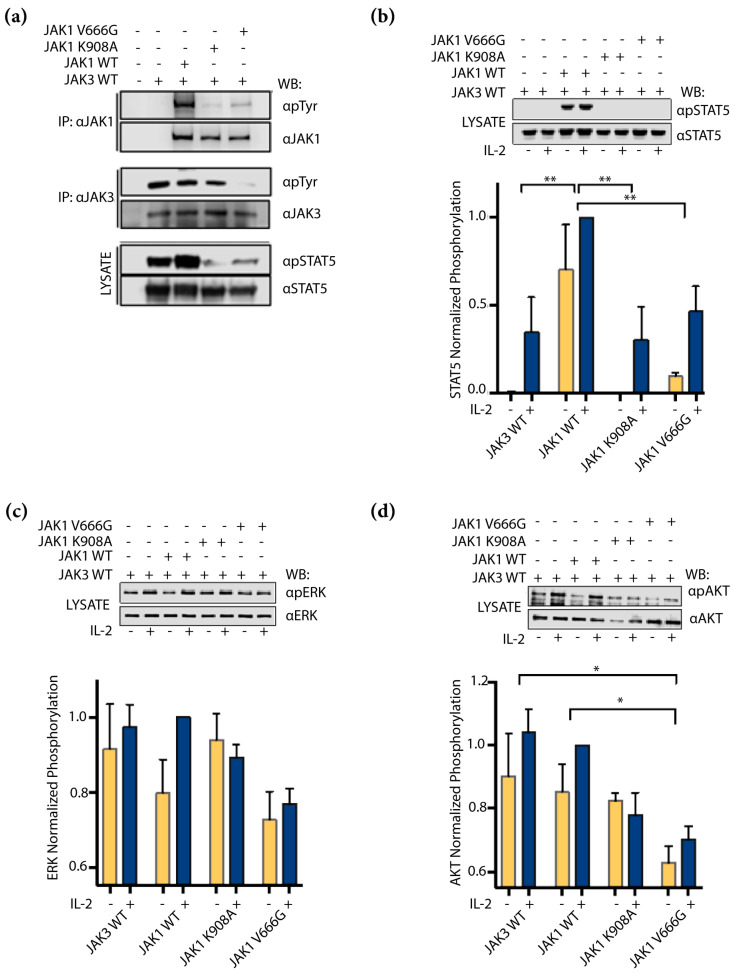
JAK1 V666G inhibits trans-activation of JAK3 and IL-2 signaling. Western blot (WB) of protein lysates from U4C cells non-transfected (NT) or transiently transfected with WT JAK1–MYC, JAK1 K908A, or V666G, along with JAK3, IL-2Rβ, γc, and STAT5. (**a**) U4C cells expressing IL-2 signaling components, including overexpressed JAK1 and JAK3, were immunoprecipitated (IP) and examined for Tyr phosphorylation by WB. Corresponding lysate was used to examine Tyr phosphorylation of STAT5. Cells were not stimulated with IL-2. U4C cells expressing IL-2-signaling components, low-level JAK1 and JAK3, were challenged with IL-2 and total cell lysate was examined for (**b**) STAT5 (*n* = 3), (**c**) ERK (*n* = 3), and (**d**) AKT (*n* = 4) phosphorylation. Graphed data include samples treated with or without IL-2 represented by normalized phosphorylation. Data were analyzed using a two-way ANOVA with a post hoc Tukey’s test for multiple comparisons. ** *p* < 0.0020 and * *p* < 0.0431.

It seemed plausible to expect that the dominant presence of JAK1 V666G on JAK3 WT protein observed in the kinase overexpression studies would affect the IL-2 signaling pathway. Whether JAK1 V666G could disrupt cell-signaling, phosphorylation via trans-activation of JAK3 and subsequent activation of STAT5, ERK and AKT downstream of IL-2, were assessed. Trans-activation under these conditions refers to phosphorylation occurring between distinct kinases facilitated by intermediate proteins such as cognate receptors. First, U4C cells were transfected with IL-2Rβ and γc to reconstitute the IL-2 receptor complex with JAK3 and JAK1 constructs. JAK1 V666G showed reduced Tyr phosphorylation compared to WT JAK1, even in the presence of JAK3 and other IL-2-signaling components (Figure 4a). In this same context, JAK3 showed a reduction in total Tyr phosphorylation when assessed in the presence of JAK1 V666G compared to cells without JAK1, or those expressing JAK1 WT and JAK1 K908A. A similar observation was seen in Tyr phosphorylation of STAT5 isolated from corresponding input lysate (Figure 4a, bottom panel). Next, the impact of JAK1 V666G on U4C cells stimulated with IL-2 to activate downstream pathways was assessed. Low levels of JAK3 and JAK1 constructs were transfected to generate a response to IL-2, because overexpressed JAK proteins were capable of auto-activation in the absence of cognate receptor or cytokine, as seen in Figure 3a–c. Consequently, transfected JAK proteins were not detectable by Western blot. Analysis of STAT5 Tyr phosphorylation was significantly reduced in samples with JAK1 K908A, and V666G compared to JAK1 WT protein (Figure 4b). ERK showed reduced, but not significant, decreases in phosphorylation with JAK1 V666G compared to samples without JAK1, or with WT JAK1 and JAK1 K908A (Figure 4c). AKT phosphorylation was significantly reduced in the presence of JAK1 V666G compared to samples without JAK1, or with WT JAK1 (Figure 4d).

### 2.5. JAK1 V666G Inhibits Tyr Phosphorylation of the JAK3 M511I- and A573V-Activating Mutants

**Figure 5:**
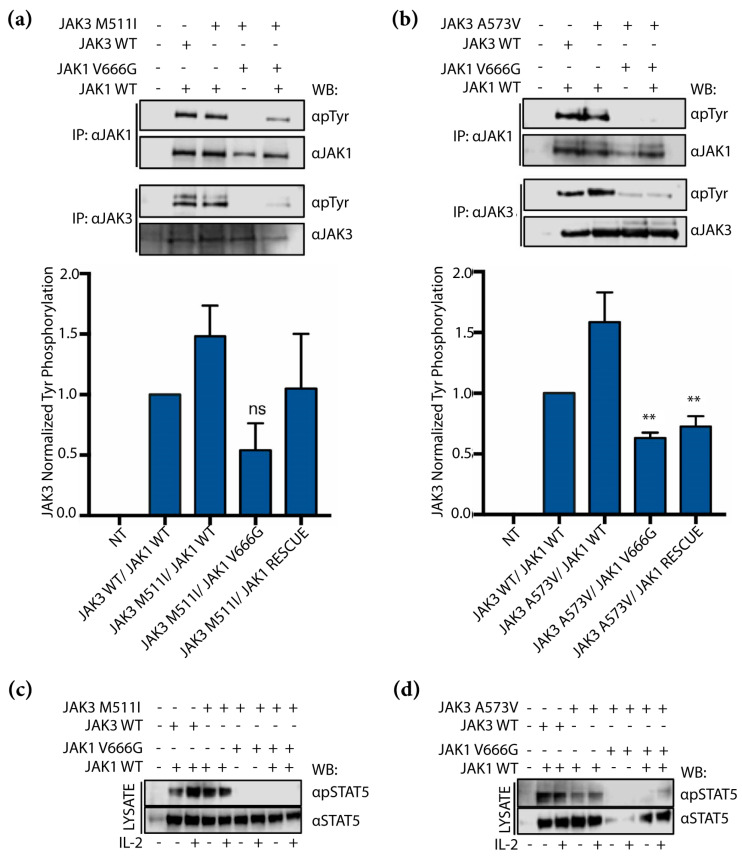
JAK1 V666G inhibits Tyr phosphorylation of JAK3 M511I and A573V activating mutants. Western blot (WB) of protein lysates from U4C cells non-transfected (NT) or transiently transfected with JAK1 MYC constructs, and (**a**) JAK3 M511I or (**b**) A573V. Overexpressed JAK1 and JAK3 were immunoprecipitated (IP) and examined for changes in Tyr phosphorylation across the transfected conditions, including a JAK1 WT rescue. Graphed data represent the means ± SEM of JAK3 M511I- or A573V-normalized Tyr phosphorylation from independent experiments (*n* = 3). Statistically significant conditions, ** *p* < 0.0073, were found using a one-way ANOVA with a post hoc Tukey’s test for multiple comparisons. ns = not statistically significant. (**c**,**d**) IL-2 receptor constructs and low levels of JAK1 and JAK3 constructs were transfected into U4C cells. Twenty-four hours post-transfection, cells were stimulated with IL-2 for 10 min. Total cell lysate was examined for STAT5 phosphorylation.

As discussed previously, JAK3-activating mutations M511I and A573V have been shown to drive T-ALL [29,30,34]. To determine whether JAK1 V666G could inhibit constitutively active JAK3 mutants, M511I and A573V, we performed co-transfections and examined their Tyr phosphorylation. JAK3 M511I and A573V showed a reduction in overall Tyr phosphorylation in the presence of JAK1 V666G (Figure 5a,b). Furthermore, the inhibitory effect of JAK1 V666G could not be rescued by the addition of WT JAK1, since Tyr phosphorylation of JAK3-activating mutants remained inhibited. Similarly, cells transfected with the same scheme as described above, but requiring low levels of JAK constructs for IL-2 inducibility, were stimulated with IL-2. JAK1 V666G disrupted STAT5 phosphorylation mediated by JAK3 M511I and A573V (Figure 5c,d). In the presence of both JAK1 V666G and JAK1 WT rescue protein, both JAK3 mutants were associated with a reduction in Tyr phosphorylation of STAT5 (Figure 5c,d). Collectively, these data suggest that constitutively active JAK3 mutants are unable to induce Tyr phosphorylation in the presence of JAK1 V666G. Lastly, JAK3-activating mutants were unable to escape the inhibitory effects of JAK1 V666G, even when combined with JAK1 WT rescue protein.

### 2.6. Structural Implications of JAK1 V666 Modeled in Open- and Closed-Kinase Confirmations

**Figure 6:**
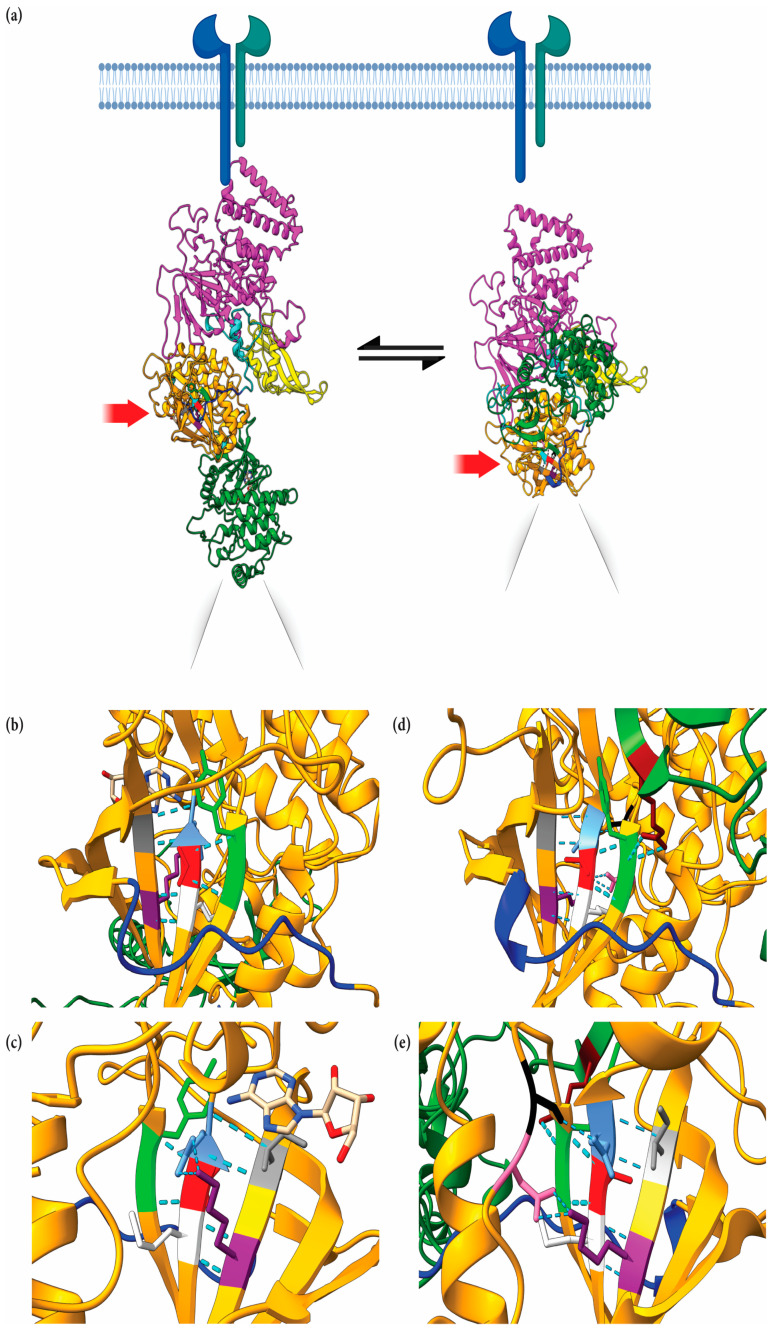
Structural implications of JAK1 V666 modeled in open and closed enzymatic configurations. (**a**) The N-terminal 4.1 protein, ezrin, radixin, moesin domain (FERM, pink), Src homology domain (SH2, yellow), pseudokinase (orange), and C-terminal kinase domain (green) are depicted within the open-kinase (left side) and closed-kinase form (right side). V666 (red) is depicted within the pseudokinase domain. Figure was created with BioRender.com (**b**) Front view of the open-kinase structure. JAK1 V666 (red) within the JH2 domain (orange), facing the SH2–JH2 linker (blue), L573–D582. JAK1 V666 forms hydrogen bonds (H-bonds; aqua dash lines) with Y654 (lime) and G655 (lime) of the anterior anti-parallel beta-sheet. (**c**) Back view of the open-kinase structure. M665 (white) and E667 (light blue) form H-bonds with K622 (purple) and I620 (grey) on the posterior anti-parallel beta-sheet. Adenosine is shown (white, red, blue stick molecule) within the JH2 nucleotide-binding pocket. (**d**) Front view of the closed-kinase structure. V666 (red) within the JH2 domain (orange) lays below the JH1 N-lobe (green), facing the SH2–JH2 linker (blue). JAK1 V666 forms singular H-bonds (aqua dash lines) with Y654, (lime) and G655 (lime) of the anterior anti-parallel beta-sheet. M665 (white) and E667 (light blue) form H-bonds with K622 (purple) and I620 (grey), respectively. JH1 (dark green) kinase domain R879 (maroon) forms two H-bonds with JH2 Y654 (lime), which forms a singular H-bond with V666 (red). (**e**) Back view of the closed-kinase structure. E667 (light blue) forms a singular H-bond with D739 (black). K622 (purple) forms two H-bonds with S738 (pink). For the sections (**c**,**e**) above, selected JH2 loops were hidden to clearly depict H-bonds. Unedited images are included as Supplementary Figure S2.

The JAK1 JH2 single-point mutation, V666G, exhibited an inhibitory effect on auto-activation, cross-activation, and IL-2 induced trans-activation. To explore the structural significance of this site on intramolecular JH1–JH2 interaction, the solved full-length mouse JAK1, bound to the cytokine receptor structure, was utilized [35] and a homology model of full-length human JAK1 was generated (Figure 6a, left side) based on the established JH1–JH2 structures [27,36,37,38]. It is likely that the mJAK1 figure by Glassman et al. [35] represents one of several forms in which JH1–JH2 domains interact [27,39]. Figure 6a depicts “open” and “closed” confirmations of human JAK1. Within the open structure (Figure 6a, left side), V666 faces the SH2–JH2 linker, residues L573–D582, (Figure 6b) to represent a key point of JAK1 dimerization, as established by Glassman et al. [35]. Hydrogen bonds (H-bonds) are predicted to form between V666 with Y654 and G655 of the anterior anti-parallel beta-sheet (Figure 6b). The JH2 nucleotide-binding site resides posterior to V666 (Figure 6c). Flanking residues, M665 and E667 (analogous kinase domain gatekeeper residues), form H-bonds with K622 and I620, respectively, on the posterior anti-parallel beta-sheet, presumably to stabilize the JH2 analogous “active site”. Conceivably, mutation of V666 would disrupt nucleotide binding to JH2 by altering these interactions and maintaining the configuration. Of note, JAK family JH2 domains can bind ATP and may provide kinase stability [40], but are not necessarily involved in phosphorylation events, with the exception of JAK2 [41].

Within the homology model of the closed human JAK1 structure (Figure 6c, right side), the V666 residue is adjacent to the JH2 analogous active site, facing the SH2–JH2 linker (Figure 6d), and positioned beneath the JH1 N-lobe (Figure 6e). In this form, V666 maintains H-bonds with Y654 and G655 (Figure 6d). Similar to the open form, the V666 flanking residues, M665 and E667, form H-bonds with K622 and I620 on the posterior anti-parallel beta-sheet, respectively. Interestingly, the JH1 kinase domain R879 forms two H-bonds with JH2 Y654, which itself forms a H-bond with V666 (Figure 6d). A back view shows that E667 forms a singular H-bond with D739 (Figure 6e) and no longer forms H-bonds with I620, as visualized in the open form (Figure 6b,c). In this state, K622 forms two H-bonds with S738 (Figure 6e). Within this structural confirmation, V666 is located near hypothetical salt bridges, proposed by Cante-Barrett et al. in the pseudokinase hinge (residues 666–736) that interacts with the JH1 kinase domain loop (residues 893–904) [42]. The indirect relationship between V666 and R879, seen in Figure 6d, may be critical for stabilizing the pseudokinase–kinase interface. It is tempting to speculate that the V666G mutation may alter the interactions within this region.

## 3. Discussion

JAK1 is an essential mediator of IL-2 receptor signaling. However, it is unclear whether the absence of JAK1 or the presence of inactive JAK1, either as a classical JH1 kinase-dead mutant (JAK1 K908A) or via JH2 mutation, would affect JAK3 activation and IL-2 signaling. Deciphering these outcomes could provide support for trans-inhibition between JAK kinases. In agreement with earlier reports [33,43], we demonstrate that inactive JAK1 JH1 and JH2 mutants can prevent IL-2-induced activation of STAT5 (Figure 4b) [43,44]. However, the data also suggest that in the absence of JAK1, JAK3 can sustain IL-2 signaling, evidenced by its phosphorylated form (Figure 4a) and downstream phosphorylation of ERK and AKT (Figure 4c,d) that can be reduced when co-expressed with JAK1 V666G. Perhaps, JAK3 alone is capable of phosphorylating IL-2Rβ and can be disrupted by JAK1 V666G.

JH2-activating JAK3 mutants, including A573V and M511I, have been reported to require IL-2 receptor and JAK1 for their transforming ability [44]. Previous reports of JH2-“inactivated” mutant JAK1 observed reduced STAT5 basal activity, even in the presence of JAK3 R657Q-tactivating mutant [43]. Here, JAK1 JH2 mutant V666G disrupted the phosphorylation of ALL-associated JAK3 M511I, A573V, and downstream STAT5. Others have demonstrated this effect with traditional JH1 kinase-dead mutant, JAK1 K908A [44]. Here, the same effect was observed with the JH2 kinase-dead-like mutant, JAK1 V666G (Figure 5). Lastly, a rescue JAK1 WT protein present with JAK1 V666G does not ameliorate the reduced phosphorylation of JAK3 M511I or A573V (Figure 5). It is unknown whether the oncogenic STAT5 N642H found in this same patient is susceptible or impervious to the inhibitory effects of JAK1 V666G.

Furthermore, it is unclear whether the inactivating JAK1 V666G mutation presents a selective advantage in ALL. One possibility could be that JAK1 V666G inactivation helps subvert detection by the immune system. For example, cancers with high mutational burden have JAK1 loss-of-function mutations [45]. The negative regulator, suppressor of cytokine signaling-1 (SOCS1), is induced by many cytokines [46] that depend on JAK1. We speculate that decreased SOCS1 expression from JAK1-inactivating mutations is advantageous for cancers, based on reports of the association between SOCS1 and p53 activation [47]. An alternative possibility is that the expression of JAK1 V666G is decreased or perhaps degraded in vivo. Indeed, the expression of total JAK1 V666G in the U4C system, utilized herein, was consistently lower than that of WT JAK1. Taken together, JAK1 V666G was not necessarily found to be an “activating mutation”, yet the investigation of the V666 region revealed that JAK1 can regulate its own, as well as other JAK-catalytic activities.

Structural studies of JAK kinases [48] make it tempting to speculate the inhibitory effects of JAK1 V666G in cis-interaction between intramolecular JH2 and JH1 domains. Within the “closed” kinase structure, JAK1 V666G is located near hypothetical salt bridges, as proposed by Cante-Barrett et al. in the pseudokinase hinge (residues 666–736) that interacts with the JH1 kinase domain loop (residues 893–904) [42]. Therefore, the mutation from valine to glycine within the hinge may promote flexibility, and subsequently strengthen the electrostatic inhibitory interactions between the JH2 and JH1 interface. Second, current evidence suggests that ATP binding within the JH2 of the JAK family members provides structural stability, enhancing JH1 activity [40,43,49]. Perhaps, V666G disrupts ATP binding and compromises JH1 stability, resulting in an inactive form of the JAK1 kinase. It was additionally demonstrated that typical kinase inhibitors target residues flanking the hinge of canonical kinase domains [50], and that maybe this region could be similarly targeted to allosterically inhibit JAK1 kinases, along with other JAK family members not mutated or tested herein. These latter interpretations alone do not account for the inhibitory effects of JAK1 V666G on partner JAKs, which suggest a trans-inhibitory mechanism.

Previous models of JAK1 from partial JH2–JH1 proposed JH2 trans-inhibition suggest that dimerized JAKs interact between JH2 and JH1 domains, where the JH2 domain of one JAK inhibits the JH1 domain of the other JAK [51,52]. V666 is localized within the hinge of the JH2 near the hub of activating mutants, notably V658F (analogous to JAK2 V617F). As proposed by Brooks et al. [53], the V617F “hotspot” and the activation loop in JAK2 JH2 is predicted to trans-interact with the active site of another JAK2 JH1. Perhaps, the V666G mutation strengthens a similar interface between the JH2 of JAK1, by altering its activation loop and interaction with the JH1 of JAK3. One residue flanked to V666 is the JH2 gatekeeper, E667 [54], and this hallmark residue can influence conformational repositioning of the activation loop [55]. Thus, we postulate that V666G promotes an activation loop projected outward from the JAK1 JH2, enhancing its interaction with the active site of the JAK3 JH1 domain. In addition to JAK3, JAK1 V666G also disrupted Tyr phosphorylation of WT JAK1 and JAK2, suggesting that inhibitory contact sites may exist between these kinases as well.

To date, FDA-approved JAK kinase inhibitors primarily target the ATP-binding pocket in JH1 domains [23], but are not approved for the use in leukemia. Thus, drug discovery efforts focused on mechanisms to inhibit JAKs by allosteric regulation are necessary. Efforts aimed towards JAK inhibition are not limited to the treatment of cancer, as they also have roles in treating atopic dermatitis, myeloproliferative neoplasms, and inflammatory disorders [17]. It is tempting to speculate that structural studies on JAK JH2/JH1 trans-interactions are required to better define the physical contacts between JAK1 V666G and JAK3. This interface likely forms outside the JAK ATP-binding pocket and may be exploited by new drugs to mimic this allosteric inhibition. In closing, these findings support trans-inhibitory interactions between JAK1 V666G and JAK3. Further studies on this interface are needed to exploit mechanisms of allosteric inhibition and provide new avenues for drug discovery efforts of JAK inhibition.

## 4. Materials and Methods

### 4.1. Patient Samples, WES and Analysis

Genomic DNA was collected from a small cohort of seven healthy donor controls and nine patients diagnosed with acute lymphoblastic leukemia (ALL), as described and reported previously [31]. Briefly, the samples were collected from El Paso, TX, USA and stored at the UTEP-BBRC Tissue Biorepository. DNA was isolated using Purgene Kit A (Qiagen, Germantown, MD, USA) according to the manufacturer’s instructions, and sent for WES, with mapping of the GRCh37 genome built by Otogenetics (Atlanta, GA, USA), and then sorted by OncoMiner (UTEP-BBRC, El Paso, TX, USA) [56]. Matched samples from healthy tissue were unavailable for analysis between germline and somatic mutations. Resulting OncoMiner analytics included a protein variation effect analyzer (PROVEAN) score that predicted deleterious effects of mutations on protein function with a recommended cut-off value of −2.5 [57], and was used to identify relevant mutations. OncoMiner results from individuals were combined and used to determine the number and type of single-nucleotide polymorphisms (SNPs) across the samples. The JAK/STAT-pathway-related gene (γc-linked receptors (γc –LR), JAK, STAT, SHIP1, and SOCS) SNPs identified within each specimen were tabulated and presented in a heatmap, using ggplot2 in R.

### 4.2. Plasmids and Site-Directed Mutagenesis

The human JAK1 MYC tag (Origene, RC213878, Rockville, MD, USA), JAK1 GFP tag (Origene, RG213878), JAK2 (Origene, RC220503), and JAK3 (Invitrogen pcDNA3.1) plasmids were purchased. IL-2Rβ, γc, STAT5B, and mutant M511I and A573V JAK3 were generated and reported previously [34,58]. Mutant forms of JAK members were prepared using the QuikChange XL site-directed mutagenesis kit (Agilent, Santa Clara, CA, USA), according to the instructions of the manufacturer. Primers used for JAK family mutants are indicated as follows: JAK1 V666G forward primer, 5′-CGTGGAGAATATCATGGGGGAAGAGTTTGTGGAAG-3′; JAK1 V666G reverse primer, 5′-CTTCCACAAACTCTTCCCCCATGATATTCTCCACG-3; JAK1 K908A forward primer 5′AGGGGAGCAGGTGGCTGTTGCATCTCTGAAGCCTG3′; JAK1 K908A reverse primer, 5′CAGGCTTCAGAGATGCAACAGCCACCTGCTCCCCT3’; JAK3 M511I forward primer 5′-CCAATACCAGCTGAGTCA GATCACACACAAGATCCCTG-3, JAK3 M511I reverse primer, 5′-GAGGGATCTTGTGAAATGTGATCTGACTCAGCTGG TATTGG-3′; JAK3 A573V forward primer, 5′-GTCATTCCTGGAAGCAGTGAGCTTGATGAGCCAAG-3′, JAK3 A573V reverse primer, 5′-CTTGGCTCATCAAGCTCACTGCTTCCAGGAATGAC-3′. All subclones and mutations were verified by Sanger sequencing of plasmids, performed at the Biomolecule Analysis and Omics Core Facility of the Border Biomedical Research Center (BBRC) at UTEP, El Paso, Texas.

### 4.3. Cell Culture, Transfections for Auto-Activation, Cross-Activation and IL-2 Trans-Activation Experiments

The JAK1-deficient human U4C cell line (ATCC) was grown in DMEM, supplemented with 10% fetal bovine serum (FBS, Atlanta Biologicals), 2 mM L- glutamine (Corning, Corning, New York, NY, USA), and 1% penicillin/streptomycin (Corning). Cells used for non-cytokine transfection experiments received complete media, while cells used for cytokine experiments were made quiescent by receiving 1% FBS media, and incubated for 24 h post-transfection. When cytokines were used, cells were stimulated with 400 IU/mL rhIL-2 for 10 min at 37 °C, prior to immunoprecipitation and Western blot analysis.

U4C cells were transfected according to the manufacturer’s instructions (Lipofectamine 2000, Invitrogen, Waltham, MA, USA). For all auto-activation and cross-activation studies, cells were transfected with 15 μg of indicated JAK plasmid, along with 10 μg of STAT5B. For auto-activation experiments, MYC-tagged JAK1 WT or JAK1 constructs, K908A or V666G, were transfected with STAT5B and used for overexpression and in vitro kinase assays. Briefly, cells were harvested 24 h post-transfection and subsequently pelleted, lysed, and clarified, as described previously [59]. A sample of input total cell lysate was saved prior to immunoprecipitation of remaining volume with anti-JAK1 (Santa Cruz, sc376996, Santa Cruz, CA, USA). For kinase reactions, immunoprecipitated protein was either incubated in kinase buffer (20 mM Tris-HCl, pH 7.5, 5 mM MgCl_2_, 5 mM MnCl_2_) alone, or supplemented with 100 μM ATP at 32 °C for 20 min, as previously described [59,60]. All protein samples were subsequently Western blotted to establish activation. 

Similarly, for cross-activation experiments, cells were transfected to overexpress the JAK1 WT GFP tag, JAK2 WT, or JAK3 WT with STAT5B, either alone or with the JAK1 WT MYC tag, or JAK1 MYC tag constructs, K908A or V666G. Samples were immunoprecipitated with either Origene antibody against GFP tag (TA150041), or Santa Cruz antibodies against c-MYC (sc40), JAK1 (sc376996), JAK2 (sc390539), or a polyclonal antibody generated against the carboxyl terminus of JAK3, described previously [60].

For IL-2 signaling experiments, U4C cells were transfected with 5 μg IL-2Rβ, and 5 μg γc to reconstitute IL-2 receptor. For Figure 4a, this system included 15 μg JAK3 WT expressed alone, or with JAK1 MYC constructs (WT, K908A and V666G) and STAT5. Input total cell lysate was saved prior to immunoprecipitation. Figure 4b–d transfections were performed to examine the effects of JAK1 V666G on IL-2-induced activation of STAT5, ERK, and AKT. This system included the same experimental scheme described above with JAK1 MYC constructs and JAK3, adjusted to 500 ng to avoid JAK auto-activation, incapable of responding to IL-2.

Hyperactive JAK3 mutation experiments included 15 μg of JAK3 WT, JAK3 M511I, or JAK3 A573V, expressed with either JAK1 MYC WT or V666G for immunoprecipitation. Complementary to this scheme, a separate set of experiments used for total cell lysate, was examined for IL-2-induced activation of STAT5.

### 4.4. Western Blot Analysis

Samples were separated by 7.5% or 10% SDS-PAGE, and transferred to a polyvinylidene difluoride membrane (MilliporeSigma, Burlington, MA, USA) for Western Blot analysis, as previously described [59,60]. For immunoprecipitation experiments, membranes were probed overnight with mouse monoclonal anti-phosphoTyr (αpTyr) antibody (4G10, EMD Millipore) and developed using horseradish peroxidase-conjugated goat anti-mouse, and visualized by chemiluminescence using LI-COR and the Image Studio Lite software. Membranes were stripped and re-probed for JAK1 (sc295, Santa Cruz), JAK2 (06-1310, Millipore) or JAK3 (ab78116-100, Abcam, Cambridge, UK). Similarly, for lysate experiments or when indicated, membranes were probed for pTyr STAT5 (05-495, Millipore), phosphorylated extracellular-signal-regulated kinase (pERK) (Cell signaling, 4370S, Danvers, MA, USA), or phosphorylated protein kinase B (pAKT) (Cell signaling, D25E6), then stripped and re-probed for total STAT5 (610191, BD-Transduction), ERK (Cell singling 4695) or AKT (Cell signaling, C67E7). Using the Image Studio software suite (LI-COR), densitometry was performed on Western blots of phosphorylated and total protein, where ratios were normalized to WT protein controls.

### 4.5. JAK1 Modeling

The open and closed structures of wild-type human JAK1 were developed by aligning the crystallographic structure of mouse JAK1 (PDB ID: 7T6F) and human TYK2 domains (PDB ID: 4OLI) as templates, respectively. Swiss-Model was used to diminish clashes and minimize the energy for the final structures. All molecular graphics and analyses were performed with UCSF Chimera [61]. BioRender.com was used to create images.

### 4.6. Statistical Analysis

One-way analysis of variance (ANOVA), followed by a Tukey’s multiple comparison test, were performed and graphed using the GraphPad Prism version 7.00 for Mac OS X, GraphPad Software, www.graphpad.com. Similarly, two-way ANOVA and a Tukey’s multiple comparisons were used under the guidance of the UTEP BBRC Statistics Core team for analysis.

## Data Availability

Genomic data were previously generated, shared and made accessible through the UTEP Bioinformatics Repository https://datarepo.bioinformatics.utep.edu/getdata?acc=B4U5YED0W0RIE3C.

## Supplementary Materials

The following supporting information can be downloaded at: https://www.mdpi.com/article/10.3390/ijms24076805/s1.
