# Supplementary material for: JAK1 Pseudokinase V666G Mutant Dominantly Impairs JAK3 Phosphorylation and IL-2 Signaling
Source: Int J Mol Sci. 2023 Apr 6;24(7):6805. doi: 10.3390/ijms24076805 (PMC10095075; doi:10.3390/ijms24076805)
Supplement: Supplementary file 1 [file ijms-24-06805-s001.zip › ijms-2237759-supplementary.pdf]

(a)

GENE	JAK1
CONTROL	0
ALL	1
PROVEAN	-6.846
AA FROM	V
RESIDUE	666
TO AA	G
DOMAIN	JH2

(b)

JAKs				*			
JAK1	N	I	M	**V**	E	E	F
JAK2	N	I	L	**V**	Q	E	F
JAK3	T	-	M	**V**	Q	E	F
TYK2	N	I	M	**V**	T	E	Y

SPECIES				*			
HUMAN	N	I	M	**V**	E	E	F
MOUSE	N	I	M	**V**	E	E	F
ZEBRAFISH	N	I	M	**V**	E	E	F
RAT	N	I	M	**V**	E	E	F
CHIMP	N	I	M	**V**	E	E	F
CARP	N	I	M	**V**	E	E	F
COW	N	I	M	**V**	E	E	F
DOG	N	I	M	**V**	E	E	F
CHICKEN	N	I	M	**V**	E	E	F
CHAMELEON	N	I	M	**V**	E	E	F
FROG	N	I	M	**V**	E	E	F
PUFFER-FISH	N	I	M	**V**	E	E	F

Figure S1. JAK1 V666G mutation in P1 pre-T ALL-patient sample. (**a**) P1 contained an unreported JAK1 psuedokinase (JH2) valine (V) 666-to-glycine (G) heterozygous mutation, which had a PROVEAN score of −6.846, and a variant allele frequency (VAF) score at 20% . (**b**) JAK1 V666 (indicated by *) is conserved amongst JAK family members and across various species. -3 amino acids and +3 amino acids surrounding V666 are shown.

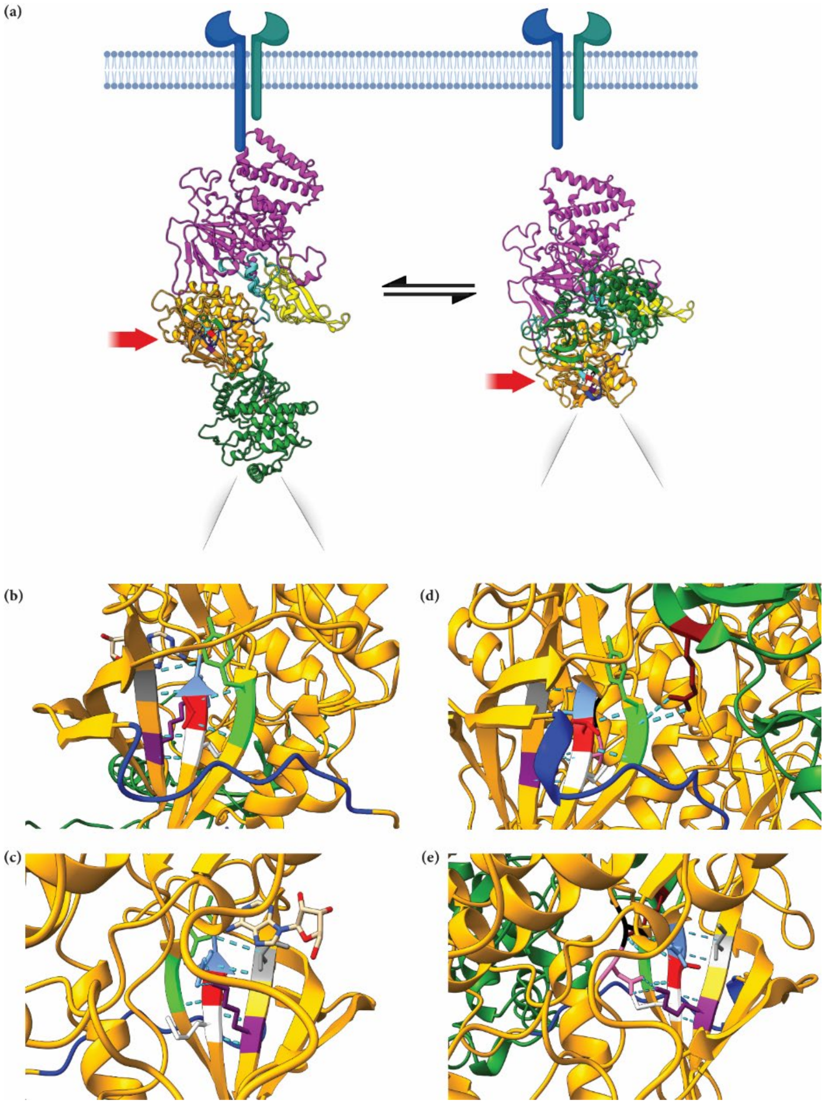

Figure S2. Structural implications of JAK1 V666 modeled in open and closed enzymatic configurations. (**a**) The N-terminal 4.1 protein, ezrin, radixin, moesin domain (FERM, pink), Src homology domain (SH2, yellow), pseudokinase (orange), and C-terminal kinase domain (green) are depicted within the open-kinase (left side) and closed-kinase form (right side). V666 (red) is depicted within the pseudokinase domain. Figure was created with BioRender.com (**b**) Front view of the open-kinase structure. Identical to depiction in Figure 6b. (**c**) Back view of the open-kinase structure. Anterior JH2 loop (orange) was deleted to enable a clear view of H-bonds depicted in Figure 6c. (**d**) Front view of the closed-kinase structure. Identical to depiction in Figure 6d. (**e**) Back view of the closed-kinase structure. Anterior JH2 loop (orange) was deleted to enable a clear view of H-bonds depicted in Figure 6e.
